# Treatment of Dead Teeth

**Published:** 1875-10

**Authors:** R. R. Freeman


### TREATMENT OF DEAD TEETH.

RY R. R. FREEMAN M. D., D. D. S.

*Read before the Tennessee Dental Association.*

Advanced operative dentistry has more consideration for the living than the dead. Their great aim is preservation of life and restoration to health. You will find for the last few years voluminous and exhausted essays directing the practitioners how to save exposed pulps. Numberless modes are set forth as to how this may be done, and the advocates of each are zealous in their views, claiming almost unlimited success in their peculiar method. It is to be recognised that great advances have been made, and many teeth are preserved to-day, pulps and all, the attempt at which has been heretofore considered useless, notwithstanding our attainments and the dissemination of knowledge among the people, we are called upon daily to treat dead teeth rendered so by neglect, accident, or maltreatment.

The death of a pulp may arise from numerous causes; that most frequent being the penetration of caries into the pulp cavity permitting the contact of irritating and extraneous substances, among other causes may be enumerated, irritation from thermal changes.

Inflammation induced by mercurial poisoning or salivation, mal-articulation inefficient nutrition, a too vigorous use of the mallet, heroic wedging etc. Under certain constitutional conditions of a patient, a large filling may induce the death of a pulp when under more favorable circumstances no evil would result, a not unfrequent cause of the death of a pulp arises in our very attempt to preserve it, sufficient care not being taken to secure proper conditions before capping, also the want of knowledge as to the remedy or application indicated.

A dead tooth at best is but a frail affair. It is only retained by nature as it were by sufferance, and must sooner or later succumb.

Our best directed efforts can only look to the rendering of it as inoffensive as possible in which if we succeed it may be retained a servicable member for years.

We will for convenience divide the teeth into two classes. The anterior or those most prominent to view and the posterior or molars. We will first consider those anterior. These teeth are treated for three reasons. 1st, to relieve pain. 2d, to restore lost parts thus rendering articulation and mastication more perfect. 3d, for mere appearance.

If one of these teeth be dead and of long standing with no external opening through which gases are escaping and your object is restoration of color, you may with care complete the operation without exciting inflammation. If there be no external opening, open up the cavity by drilling through the basilar ridge directly into the pulp chamber. Let the opening be sufficient to allow reaching every portion of the canal, especially the corneau or horne like portions toward the cutting edge.

In removing the debris from the cavity do not push any of its contents through the foraman. If you do, it will be sure to excite inflammation, neither permit the instrument to pass through and wound the soft parts or trouble will be sure to follow. By careful measurement from the outside a tolerable accurate idea as to the length of the canal is obtained. In passing the broach up the canal it will strike a slight shoulder usually found at the apex of a root, thus indicating the depth you should go. This however is not an infallible guide, especially in young subjects, for the foramen, may be so large as to permit your instrument passing through without meeting this obstruction. After the canal is thoroughly cleansed and disinfected introduce a small pleget of cotton, moistened with *creosote* on a slender broach, leave the cotton in position and seal the cavity with Hill's Stopping or gutta percha to exclude extraneous substances; let this remain for a day or two, when if all is well, proceed to fill the root with gold or tin foil. I prefer the tin as I seem to be able to manipulate it more to my satisfaction. I invariably use the rubber dam when operating upon dead teeth from *first* to *last*, which when properly applied, gives full command of the situation. The next step is the restoration of the color of the tooth as nearly as possible. You need not expect to give a dead tooth its normal color, though it can be rendered much less objectionable to view. In life a tooth is supplied with a circulation of liquor sanguinious, which gives it a bright and lively appearance. In death this circulation is shut off, at least in the enamel and dentine of the crown, giving it that opaque look, the peculiar characteristic of a dead tooth.

Where a tooth has recently died, there may be considerable infiltration especially in young subjects where the dental tubuli are large. This condition may be the more easily overcome, for the same reason, owing to the largeness of the tubuli the infiltrations can be more readily removed.

Never attempt to bleach a tooth until you have the root filled, then with rubber dam applied there is no danger of injuring the soft parts. Ordinarily the excavator, warm water chloride of zinc and alcohol will remove most of the discoloration, where the red corpuscles have been forced into the dental tubuli oxalic acid is recommended to remove the hæmatine. This is used in a strong aqueous solution permitting it to remain in the cavity a few minutes, when, after securing the desired effect, wash out with warm water, then fill immediately with orychloride of zinc when this is sufficiently hardened cut away and make a gold filling. There is a certain class cases in which the slightest disturbance will cause secretion of pus, which will sometimes continue to discharge through the cavity of decay regardless of every care and treatment. In such cases cleanse thoroughly, inject iodine and creosote and fill the root at once with tin foil, then by leeching, applying iodine and aconite equal parts, or a hot roasted rosin, you may disperse the inflammation, and no farther troubles ensue. If however this will not suffice lose no time in making a direct opening through the gum down to the apex of the root you can then inject the iodine feeling assured it will reach the desired part and break up the pus secreting membrane. You may introduce a strand of floss silk into the artificial opening permitting the end to protrude a little, this will act as a seaton, when inflammation has sufficiently subsided remove the silk and permit the parts to heal.

In molar and bicuspid teeth where you can get direct entrance into the root canals the same treatment is indicated as in the front teeth.

Where there are curved and compressed roots all you can do is to make the best effort possible to cleanse and fill them. Such roots as you are satisfied you can fill completely, do so permanently, then fill the body of the tooth with Hill's Stopping, if the case remains satisfactory for a month or two then complete the permanent filling, with the assurance that the tooth may do good service for a long time to come

